# Simulating the efficacy of vaccines on the epidemiological dynamics of SARS-CoV-2 in a membrane computing model

**DOI:** 10.1093/femsml/uqac018

**Published:** 2022-09-16

**Authors:** Marcelino Campos, José M Sempere, Juan C Galán, Andrés Moya, Rafael Cantón, Carlos Llorens, Fernando Baquero

**Affiliations:** Department of Microbiology, Ramón y Cajal University Hospital, Ramón y Cajal Institute for Health Research (IRYCIS), M-607, km 9,1 28034 Madrid, Spain; Valencian Research Institute for Artificial Intelligence (VRAIN), Universitat Politécnica de Valencia, Camí de Vera s/n, 46022 Valencia, Spain; Valencian Research Institute for Artificial Intelligence (VRAIN), Universitat Politécnica de Valencia, Camí de Vera s/n, 46022 Valencia, Spain; Department of Microbiology, Ramón y Cajal University Hospital, Ramón y Cajal Institute for Health Research (IRYCIS), M-607, km 9,1 28034 Madrid, Spain; Centro de Investigación Biomédica en Red de Epidemiología y Salud Pública (CIBERESP), M-607, km 9,1. 28034 Madrid, Spain; Foundation for the Promotion of Sanitary and Biomedical Research of the Valencian Community (FISABIO), Av. Cataluña 21, 46020 Valencia, Spain; Integrative Systems Biology Institute, University of Valencia and Spanish Research Council (CSIC), Av. Cataluña 31, 46020, Valencia, Spain; Department of Microbiology, Ramón y Cajal University Hospital, Ramón y Cajal Institute for Health Research (IRYCIS), M-607, km 9,1 28034 Madrid, Spain; Centro de Investigación Biomédica en Red Enfermedades Infecciosas (CIBERINFECT), M-607, km 9,1. 28034 Madrid, Spain; Biotechvana, Valencia, CEEI Building, Valencia Technological Park., C. Agustín Escardino 9, 46980, Paterna, Spain; Department of Microbiology, Ramón y Cajal University Hospital, Ramón y Cajal Institute for Health Research (IRYCIS), M-607, km 9,1 28034 Madrid, Spain; Centro de Investigación Biomédica en Red de Epidemiología y Salud Pública (CIBERESP), M-607, km 9,1. 28034 Madrid, Spain

**Keywords:** SARS-Cov-2, vaccination, membrane computing, epidemics simulation

## Abstract

Membrane computing is a natural computing procedure inspired in the compartmental structure of living cells. This approach allows mimicking the complex structure of biological processes, and, when applied to transmissible diseases, can simulate a virtual ‘epidemic’ based on interactions between elements within the computational model according to established conditions. General and focused vaccination strategies for controlling SARS-Cov-2 epidemics have been simulated for 2.3 years from the emergence of the epidemic in a hypothetical town of 10320 inhabitants in a country with mean European demographics where COVID-19 is imported. The age and immunological-response groups of the hosts and their lifestyles were minutely examined. The duration of natural, acquired immunity influenced the results; the shorter the duration, the more endemic the process, resulting in higher mortality, particularly among elderly individuals. During epidemic valleys between waves, the proportion of infected patients belonging to symptomatic groups (mostly elderly) increased in the total population, a population that largely benefits from standard double vaccination, particularly with boosters. There was no clear difference when comparing booster shots provided at 4 or 6 months after standard double-dose vaccination. Vaccines even of moderate efficacy (short-term protection) were effective in decreasing the number of symptomatic cases. Generalized vaccination of the entire population (all ages) added little benefit to overall mortality rates, and this situation also applied for generalized lockdowns. Elderly-only vaccination and lockdowns, even without general interventions directed to reduce population transmission, is sufficient for dramatically reducing mortality.

## Teaser

Membrane computing technology simulates the effect of generalized or focused vaccination and lockdown in a town with detailed demographic complexity, experiencing an *in silico* SARS-Cov-2 epidemic over the course of 2.3 years.

## Introduction

**Figure 1.**
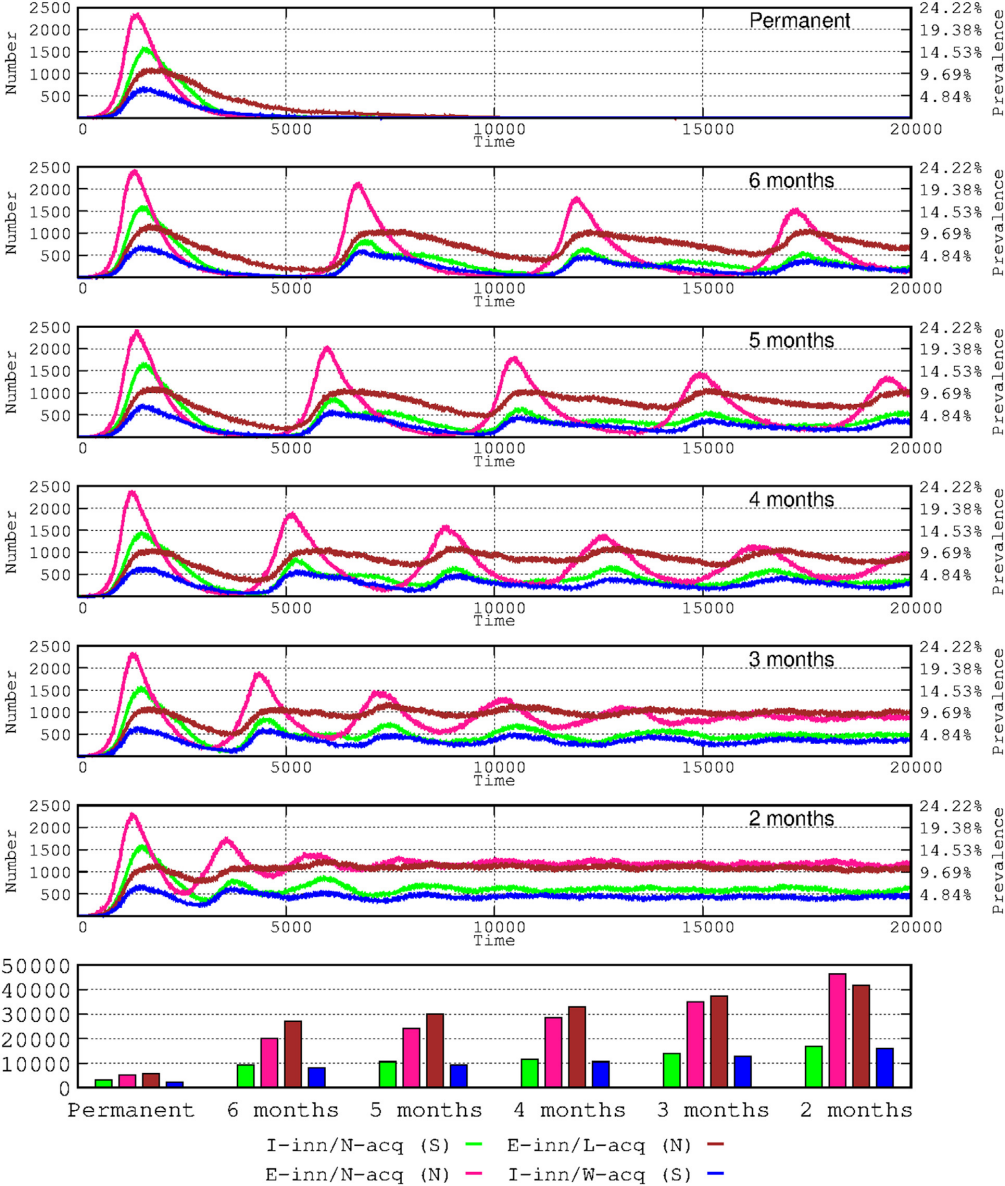
Duration of immunological protection following infection. Successive panels represent the duration of immunological protection after infection (symptomatic or not) in the number of hosts of the 4 immune-response groups in the various waves. This simulation reflects successive scenarios where the natural immune-protection is permanent or there is loss of protection 6, 5, 4, 3, and 2 months after the infection. The bar graph at the bottom of the figure reflects the total number of infected cases after 20 000 time points (2.28 years) in the 4 immune-response groups.

**Figure 2.**
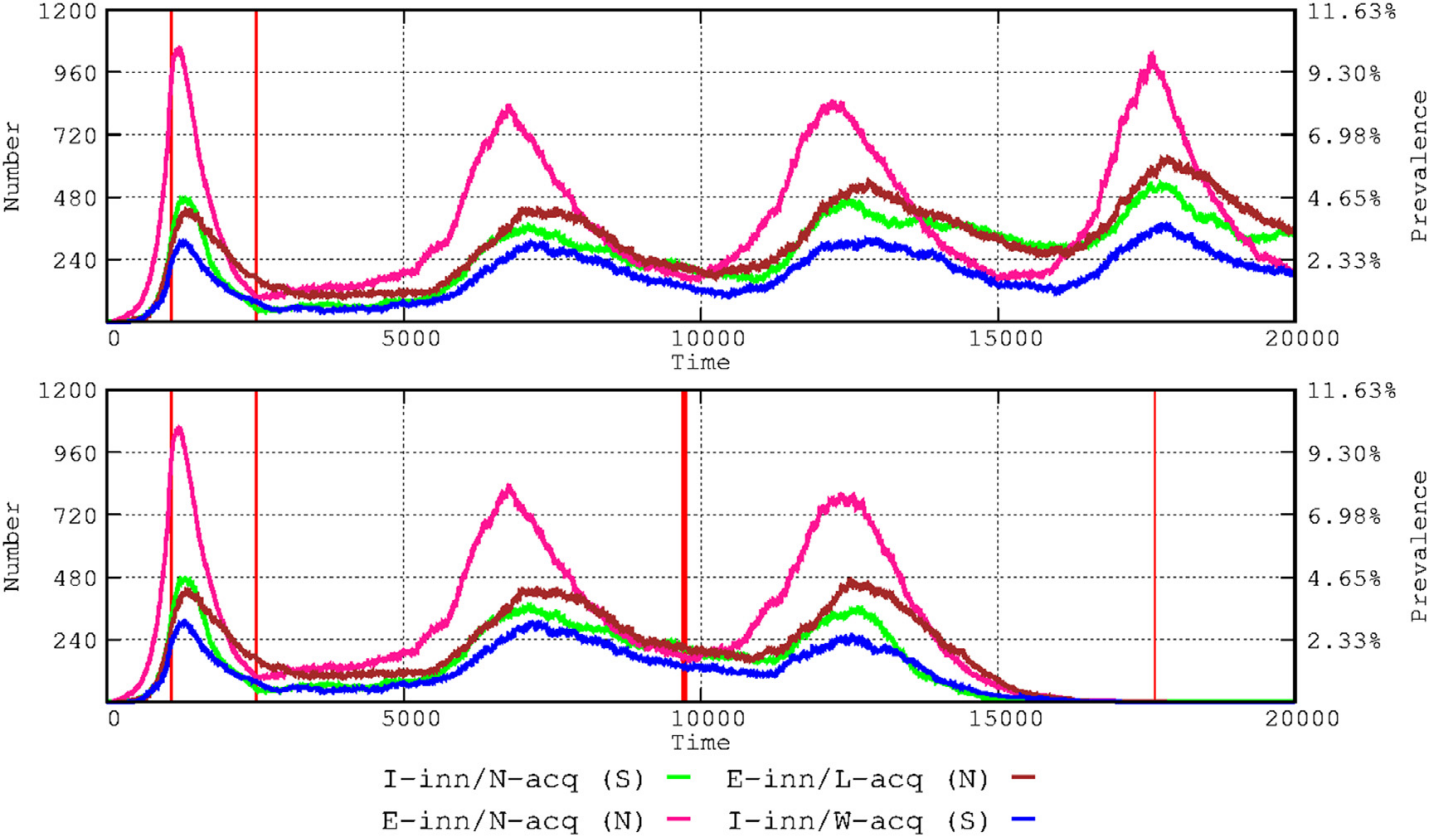
Effects of vaccination on the various immunological response groups. The upper panel shows the dynamics without vaccination; the lower panel shows the effects of a general vaccination (single dose). The thin red vertical lines at the left of each panel indicate the start and the end of the stringent lockdown period; from the end to the start of the vaccination period, less stringent interventions were adopted. In the lower panel, the red vertical thicker lines in the middle and right of the figure indicate the start and end of vaccinations (covering a full year).

**Figure 3.**
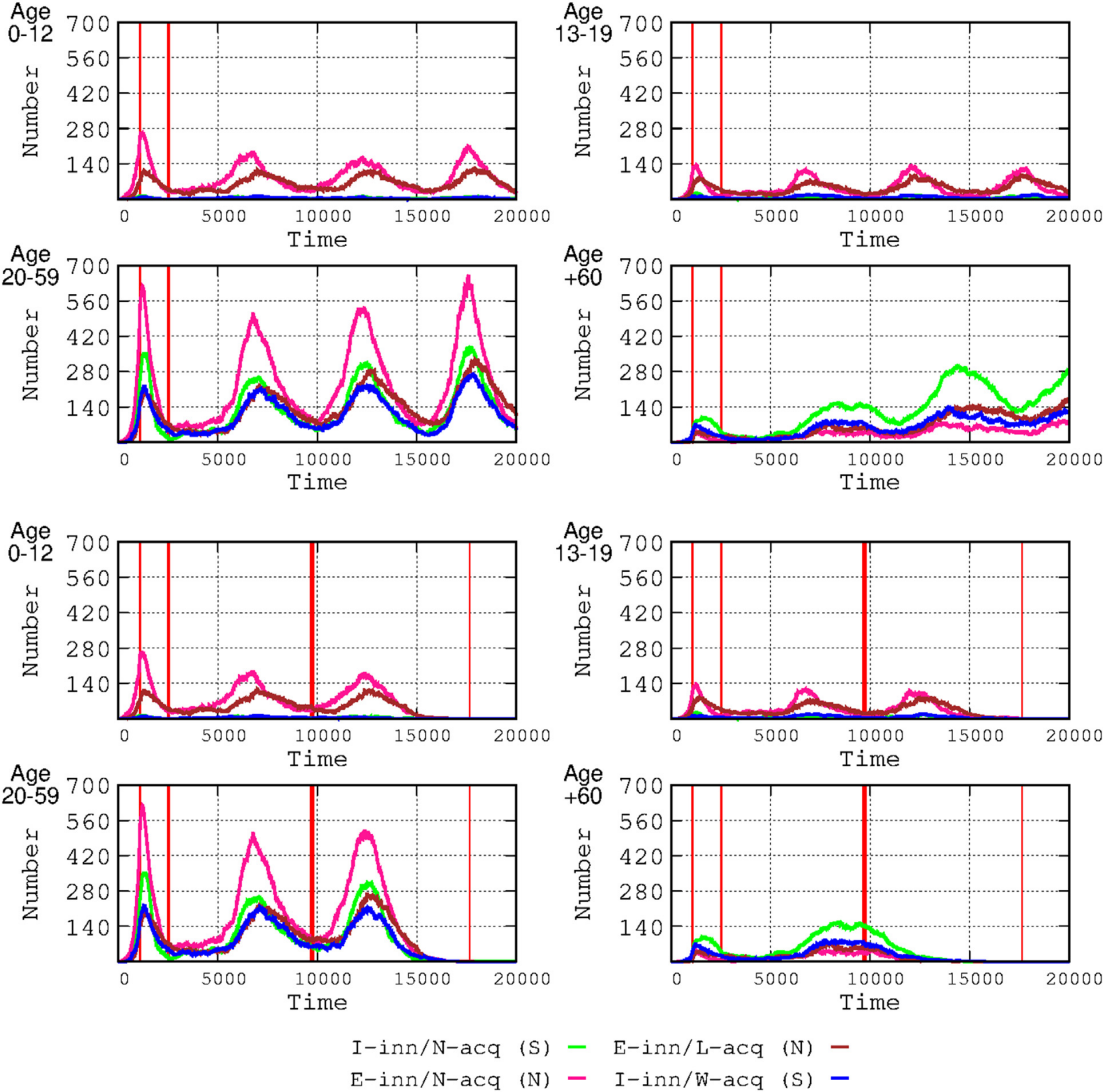
Effects of vaccination on the number of infected hosts per age group. The start of the vaccination period is marked by the thick red line, and the end (1 year later) is indicated by the thin red line. Note that, without vaccination (upper panel), the I-inn/N-acq/S (in green) immune group had a greater increase in successive waves in the adult and elderly groups (and is almost nonexistent in the children and adolescents). The vaccination had a strong immediate effect on the elderly group (who were prioritized for vaccination), aborting the third wave. The vaccination schedule prevented significant decreases in the number of infected hosts in the third wave, but the vaccination suppressed the fourth wave.

**Figure 4.**
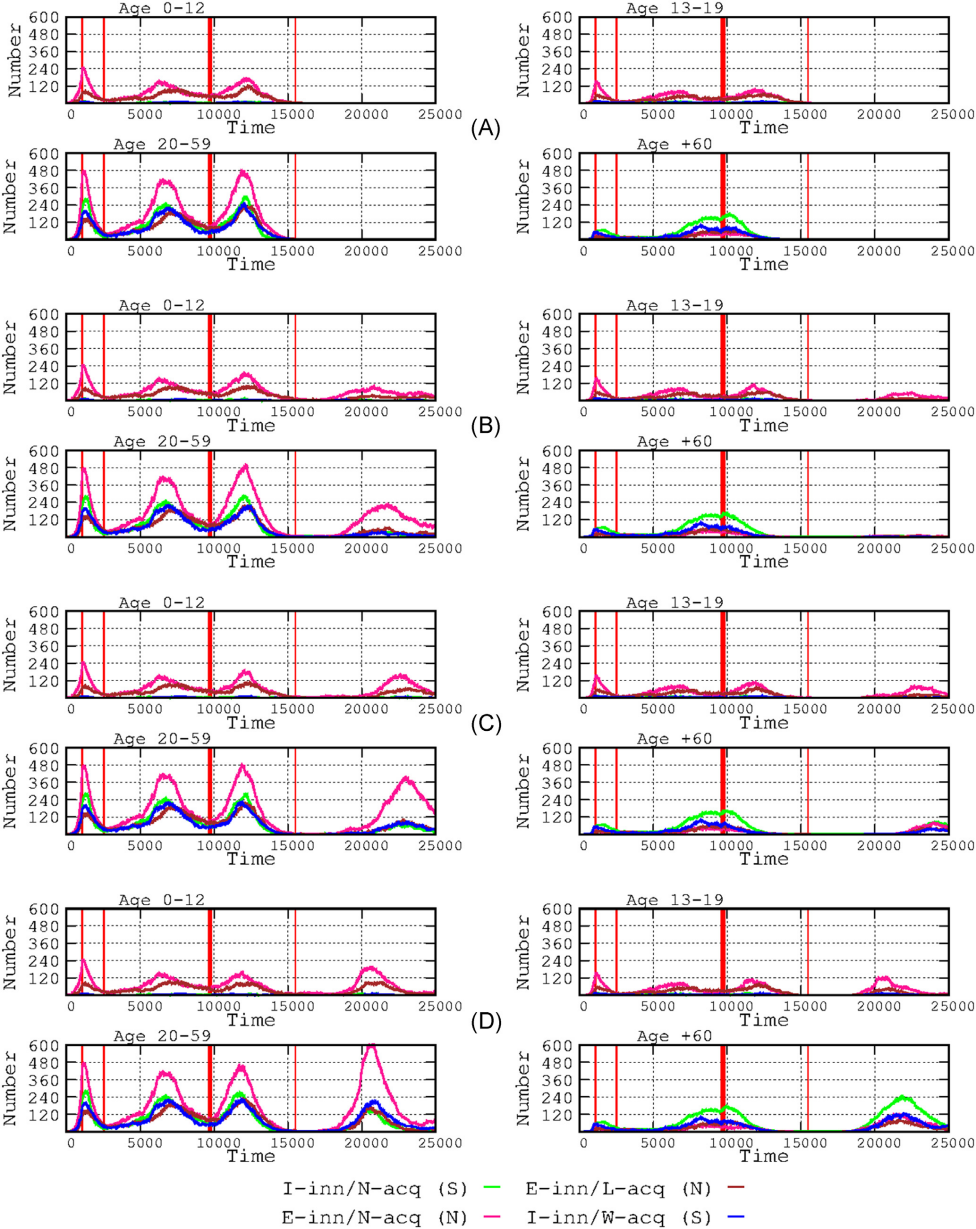
Effects of reduced vaccine efficacy over time in the number of infected cases by age group. The 4-paneled rows indicate no-loss **(A)**, moderate loss **(B)**, high loss **(C)**, and maximal loss **(D)**. The start of the vaccination period is marked by the thick red line, and the end (1 year later) is indicated by the thin red line at the right side.

**Figure 5.**
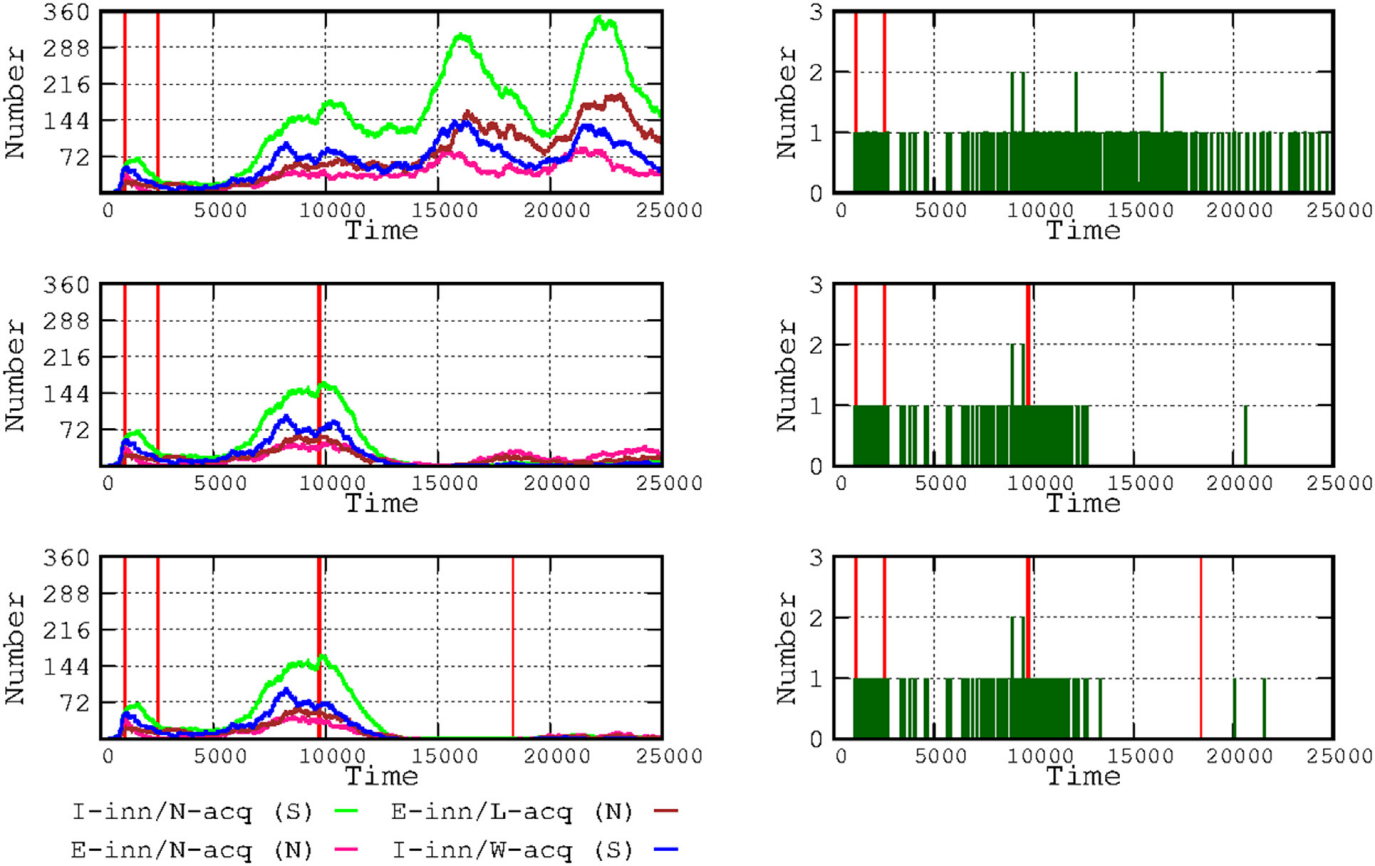
Effects of elderly-only vaccination on the number of elderly cases and mortality. On the left, the number of elderly cases; on the right, the number of deaths in the elderly group per time-step. First row, no vaccination scenario; second row, elderly-only vaccination; third row, universal vaccination.

**Figure 6.**
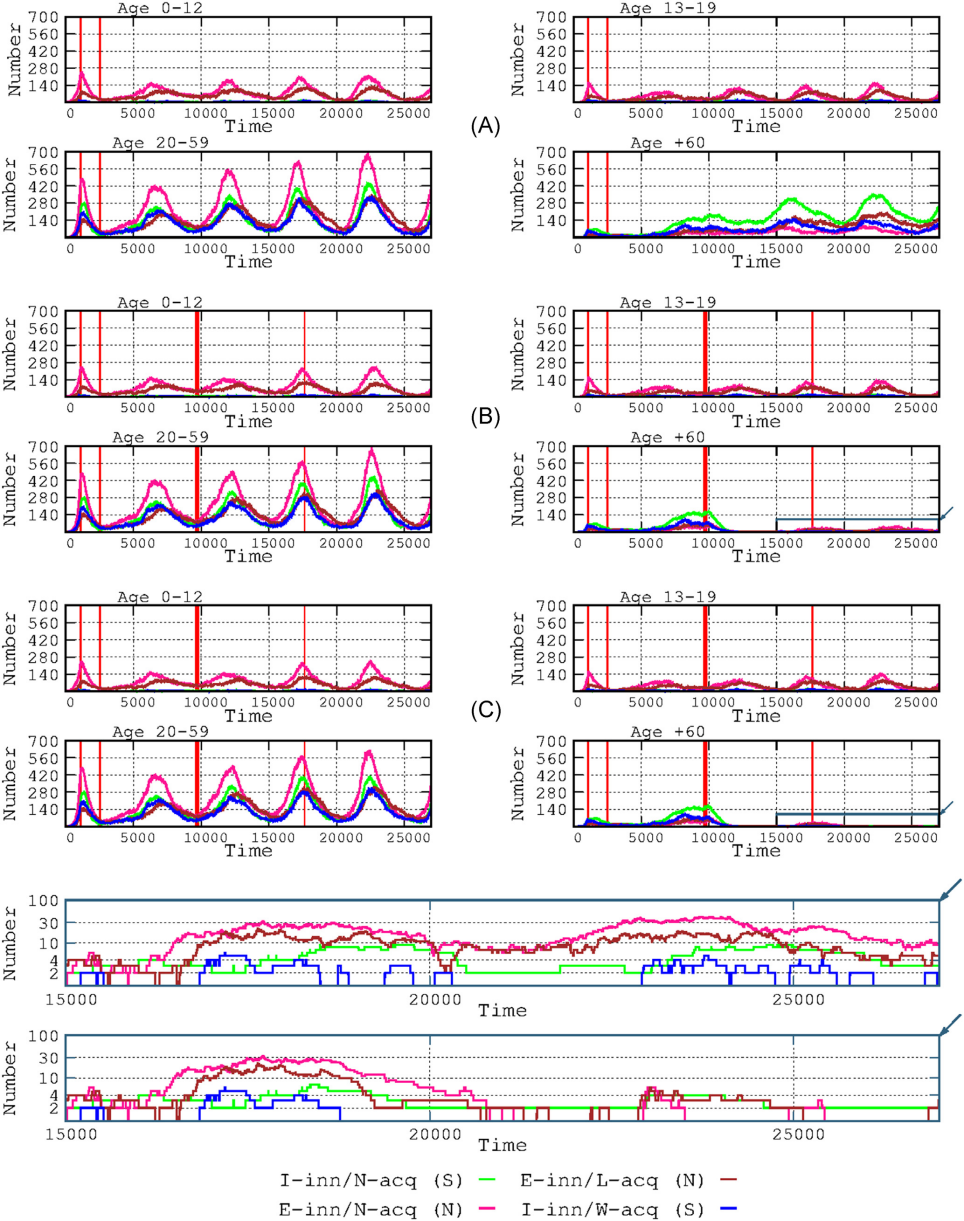
Effects of vaccination only in the elderly patient group. The influence of elderly-only vaccination on the number of infected cases in all other age groups is shown. The 4-panel ensemble at the top of the figure **(A)** shows the non-vaccination results. The two 4-panel sections below show the effects of vaccination-only in the elderly group, with and without**(B)** a booster vaccine and a third double-dose **(C)**. The bottom elongated blue rectangles detail the effects of the booster vaccine, applying a log scale. The red vertical lines delineate the various periods: from left to right, the period without any intervention, the start of the stringent lockdown, the start of the relaxed lockdown, the start of the vaccination period, and the start of the post-vaccination period. The blue boxes in the ≥60 year group **(C and D)** are represented in log scale at the bottom of the figure.

**Figure 7.**
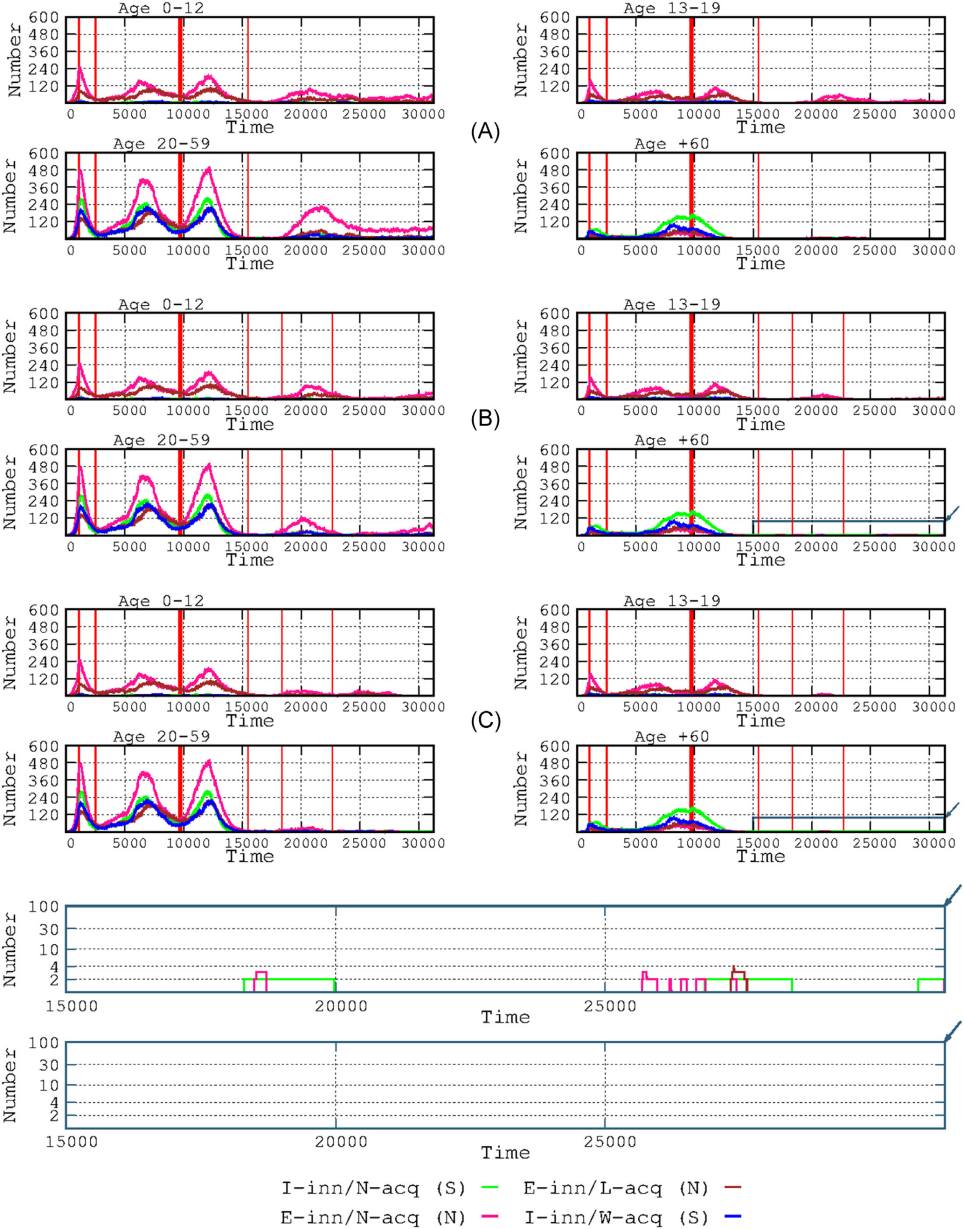
Effects of vaccine booster shots, after full vaccination was achieved by using a vaccine with moderate loss of efficacy over time. In the *x*-axis (time), the 30 000 steps (1 h each) account for approximately 3.5 years. The 3 successive 4-panel rows from the top show the effects of the absence of a booster shot**(A)**, a booster shot with a vaccine that loses protective efficacy at 6 months**(B)**, and a booster shot with maximum long-term efficacy **(C)**. The start of the vaccination period is marked by the thick red line, and the end (1 year later) is indicated by the following thin red line. The successive thin red lines represent the start and end of the boosting vaccination period.

**Figure 8.**
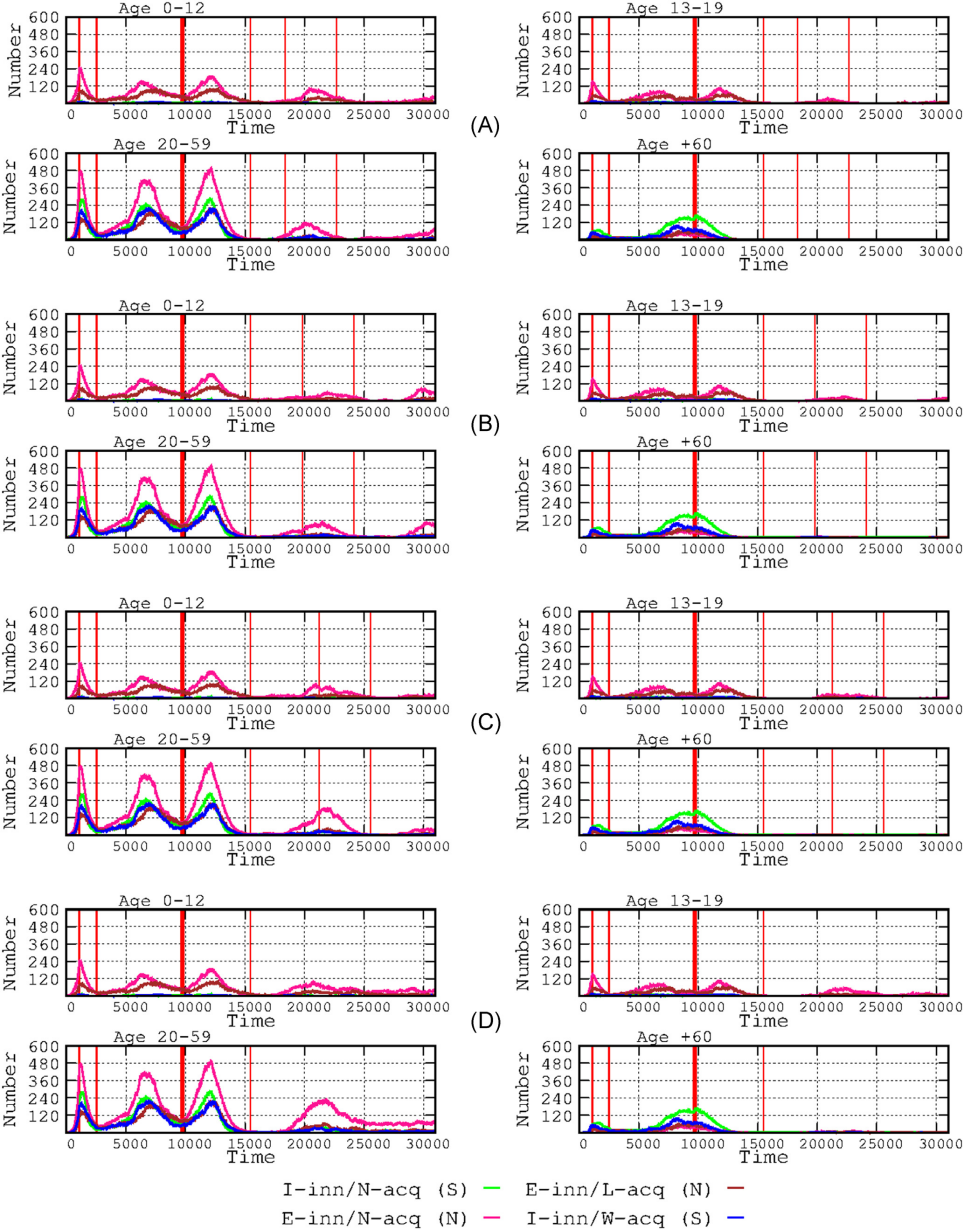
Influence of booster timing. The 4 successive 4-panel rows show the effects of the delay in administering the booster dose (4, 6, 8 months and no booster shot) after full vaccination on the number of infected hosts in the various age groups.

**Figure 9.**
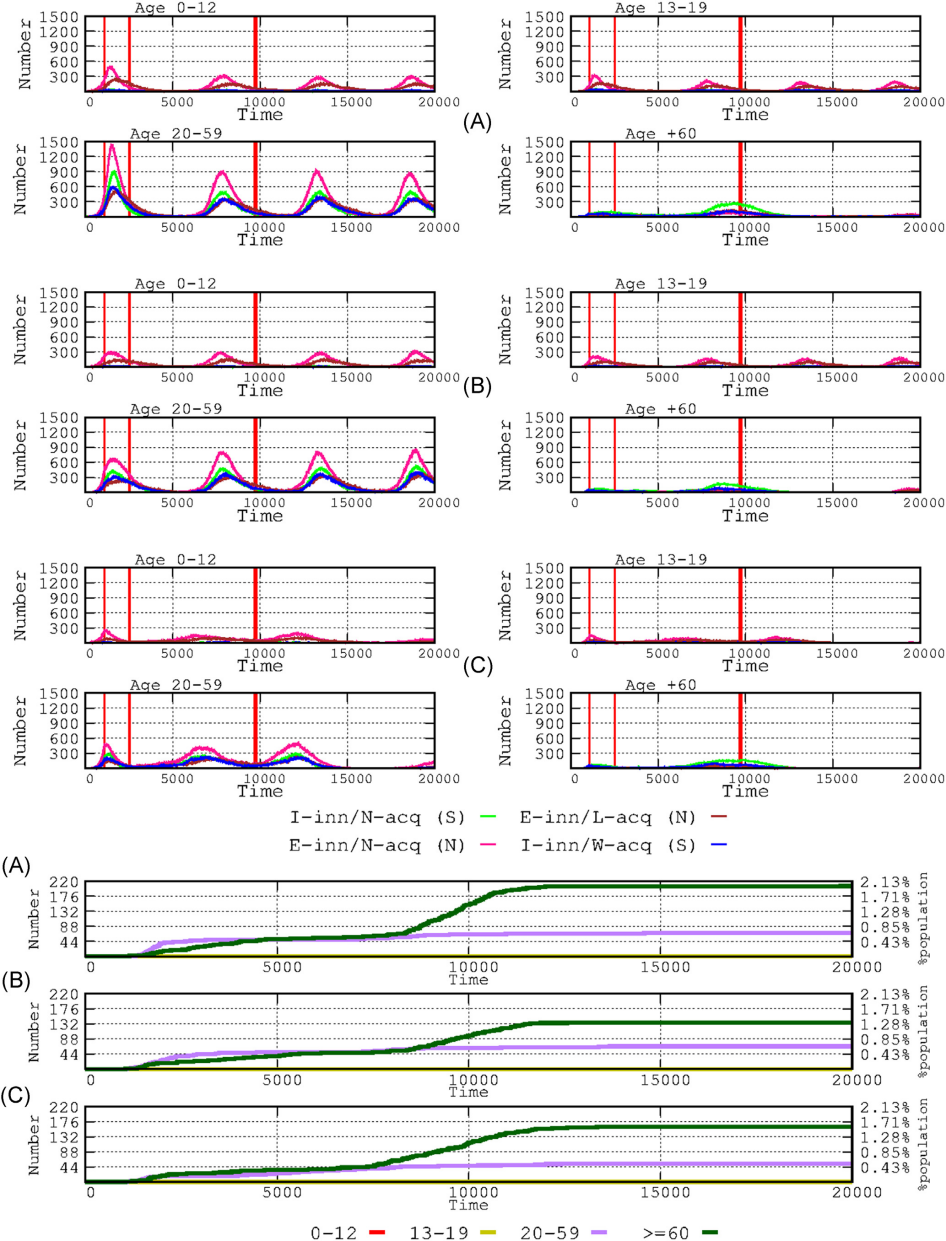
Elderly-only vaccination and elderly-only lockdown or generalized lockdown. The upper part of the figure in the 4-panel section **(A)** shows the lockdown applied only to the elderly; the vaccination schedule is maintained but with no other intervention on transmission;**(B)** lockdown only for the elderly and interventions reducing transmission are applied to the entire community (all ages);**(C)** generalized lockdown (all ages) and implementation of transmission interventions. The elongated panels at the bottom of the figure show the cumulative number of deaths in the former scenarios (A, B and C); green and violet lines correspond to the elderly and adult populations, respectively.

Membrane computing (in general cellular computing) differs from most conventional mathematical models, being able to mimic the actors of multilevel biological scenarios, which are represented by computational entities individualized by ‘membranes’ that can be submitted to the action of particular ‘objects’ (Pérez-Jiménez et al. 2003, Păun et al. 2010). In a sense, membrane computing creates scenarios in which events occur according to local conditions, probabilities, and the intensity of interactions between computational entities, resulting in predictable outcomes. In practical terms, cellular membrane computing mimics reality, which, when applied to transmissible diseases, creates a virtual ‘epidemic’ within the computational model, thereby providing insights into the effects of interventions (single or combined) and helping to make the most reasonable decisions. We have previously explored membrane computing applications in clinical microbiology in the specific field of antibiotic resistance epidemiology, considering large settings (the spread of resistance in hospitals and the community) and more precise problems (the spread of antibiotic resistance plasmids among bacterial communities) (Campos et al. 2019, Campos et al. 2020, Gil-Gil et al. 2021). The impact of non-pharmaceutical interventions on the epidemiological dynamics of SARS-Cov-2 has been recently studied using membrane computing (Baquero et al. 2021, Campos et al. 2021). In this previous study, ‘virtual’ SARS-Cov-2 was introduced in a scenario simulating a community composed of an isolated SARS-Cov-2-free population of 10 320 healthy individuals not previously exposed to the infection, which included 1372 children, 848 teenagers, 5590 adult workers, 2380 retired elderly individuals, 100 elderly individuals in nursing homes, and 30 healthcare workers. These numbers are the result of demographic adjustments; the total population size was determined according to the limitations of the computational workload. The present study employed the same basic scenario used in our previous study (Baquero et al. 2021, Campos et al. 2021). Table S1 of the supplementary material presents the demographic details, including age groups, daytime living spaces, family structure, division of the hosts’ work/school/leisure time, residence in long-term care centers, hospital stays, and intensive care unit stays. We considered the effects of natural innate and acquired immunity in the various groups of infected hosts. In this work, the sense of the term ‘innate immunity’ should be understood as ‘protected from infection’ in its original Pasteurian sense (1881). In our context, that includes not only protection by macrophages and pro-inflammatory and antimicrobial mediators, as natural antimicrobial peptides, but also by low angiotensin-converting enzyme 2 (ACE2, the natural functional receptor for coronaviruses) expression in the respiratory tract. An individual-based model (Pinotti et al. 2021) has shown that previous exposure to other coronavirus, heterotypic cross-protection, also play a role in reducing infection and clinical symptomatology. Given that most of the figures in this study represent the epidemiological dynamics of these groups, these ‘immunological response types’ are briefly summarized here and are as follows: (i) efficient innate immunity/lacking acquired immunity, with mild or no symptoms (E-inn/L-acq/N), in a proportion of children, estimated in 19% (Campos et al. 2021); (ii) efficient innate immunity/normal acquired immunity/mild, with mild or no symptoms (E-inn/N-acq/N), mostly children and adolescents; (iii) inefficient innate immunity/normal acquired immunity, symptomatic (I-inn/N-acq/S), a proportion of adults; and (iv) inefficient innate immunity/weak acquired immunity, symptomatic (I-inn/W-acq/S), mostly elderly. In Figs 1–9, the *x*-axis (time) represents hourly steps (1000 hourly steps correspond to approximately 40 days, 20 000 steps correspond to 2.28 years).

Our simulator (which is also applicable to other viral diseases) is named LOIMOS, from the ancient Greek loimos (λοιμός), meaning plague, pestilence, or any deadly infectious disorder. A user-friendly interface is being developed for LOIMOS and will be freely available. Interested readers should contact our first author, Dr. Marcelino Campos (mcampos@dsic.upv.es).

Our study includes a simulation of the natural dynamics of epidemics, considering different protection times of natural immunity, from permanent immunity to 2-month-only immunity, considering that coronavirus can produce relatively short periods of protection. In this background, the effects of vaccination (according to a likely vaccination schedule) on morbidity, mortality, and the dynamics of successive vaccination waves are presented. The effects of more or less efficacious vaccines on the epidemiological dynamics are also studied. The effects of elderly-only vaccination are then simulated, considering or not a third booster. Lastly, the combined effects of elderly-only vaccination and elderly-only or generalized interventions for decreasing transmission are simulated. The results of our computational model highlights the value of forecasting probabilistic approaches (Cramer et al. 2022) and constitute a proof of concept for predicting a wide variety of epidemiological vaccination scenarios, thereby contributing to appropriate decision-making for public health authorities.

## Results

### Developing a population immune status without vaccination

In most cases, interaction with viruses results in an immunologically protective response, which differs among the various immunological-response groups considered in the Introduction section and in our previous study (Campos et al. 2021). The progression in the number of infected hosts in each of these groups (either asymptomatic or symptomatic) is presented in Fig. 1. Permanent acquired immunity results in the extinction of the epidemic, with those hosts with asymptomatic E-inn/L-acq immunity (because of the minor viral challenge) lasting longer (brown in Fig. 1). As can be expected, the reduced time of protection for natural acquired immunity correlates with the proximity between waves and consequently with the total number of waves. The reduced duration of protection is followed by a progressive reduction (in subsequent waves) in the number of cases for most patients, the E-inn/N-acq (N) immunity group (pink), and the symptomatic I-inn/N-acq (S) immunity group (green); even the short protection of naturally acquired immunity produces such an effect. Over 5 months of protection is sufficient to produce a major reduction in the number of cases in the symptomatic I-inn/N-acq (green) and I-inn/W-acq (blue) groups. The number of asymptomatic cases in the E-inn/L-acq (mostly children) immunity group remained stable, regardless of the duration of the immunological protection.

In the long term, the cumulative number of infected cases inversely correlates with the duration of the natural protection for all 4 immunity groups. However, the cumulative number of non-symptomatic hosts presenting efficient innate immunity and normal or even low acquired immunity increases at a higher rate than for the symptomatic hosts.

Using the area under the curve for mortality along 20 000 steps (approximately 2.28 years from the start of the epidemic), we calculated the influence on mortality of the duration of natural immune protection. When the model considers the protection period lasting only 5–6 months then the mortality (total number of deaths) increased by 17.4%–18.9%. Paradoxically, shorter periods of natural immunity of 2, 3, and 4 months yielded smaller increases in mortality (11.3%, 12.1%, and 11.7%, respectively). Although mortality is likely independent of the period of natural immunity during the first wave (insufficient time to gain immunological protection), it clearly increases during the second wave, especially among the more fragile hosts and those facing difficulties for hospital care. Once the number of fragile hosts has been reduced (through death) and the full efficiency of intensive care units is restored, the increases in mortality decrease.

With successive decreases in protective acquired natural immunity, there are correspondingly more frequent waves with shorter time-intervals between them, thereby leading to a progressive curve flattening effect. Therefore, if the natural acquired immune protection lasts only for 2 months, the dynamics of the infection tend to level off, entering into a steady-state in the number of asymptomatic (pink, brown), and symptomatic (green, blue curves) patients, suggesting a progression toward an endemic landscape. The following sections analyze the effects of vaccination as if the natural acquired immunity was protecting the host for 5 months.

Abbreviations: S, symptomatic; N, mild or no symptoms; E-inn, efficient innate immunity; I-inn, inefficient innate immunity; L-acq, lacking acquired immunity; N-acq, normal acquired immunity; W-acq, weak acquired immunity.

### Simulating the effects of vaccination on epidemic waves

As previously stated, we simulated epidemiological waves by reducing the protection of acquired immunity to only 5 months. The results are shown in Fig. 2. In the absence of vaccination, 4 waves occurred along 20 000 hourly steps (2.28 years), with changes in the composition of the infected patients. The duration of the successive waves was longer than the first wave for all immunological groups, which could be the consequence of the lockdown implemented during the first wave (at step 1080, 1.5 months after the start of the epidemic). A continuous increase in the number of individuals in the E-inn/N-acq/N immunity group" (pink in Fig. 2) always preceded an increase in the other groups. The prevalence of each immunity group increased in each successive wave, particularly the predominant groups of asymptomatic and mildly symptomatic E-inn/L-acq/N (mostly children and young adults; brown line in Fig. 2) and symptomatic I-inn/N-acq/S (mostly adult and elderly groups, green line). During the troughs between the waves, the proportion of infected patients belonging to the symptomatic groups increased.

Our epidemic simulation started with a 1.5-month period without interventions, followed by a 2-month stringent lockdown, and lastly 10 months of a less stringent lockdown (see Materials and Methods section). The vaccination started on time-step 9720 (13.5 months after the start of the epidemic) and consisted of 2 doses per host, separated by 3 weeks. The vaccination was implemented in descending order of age, prioritizing the elderly and adult population (see Materials and Methods section). The entire population was vaccinated at 1 year from the start of the vaccination schedule. The vaccination strongly affected the prevalence of symptomatic patients (mostly adults and elderly hosts) and, as expected, affected the prevalence of younger hosts only at a later stage only lately the younger hosts. In our simulation, the emergence of new waves was completely prevented (Fig. 3).

### Effects of vaccines with various levels of efficacy over time

Figure 4 presents the results for the influence of the varying degrees of loss of vaccine efficacy over time ranging from moderate to maximum (see definitions in the Materials and Methods section), in the various age groups and immunological response groups. Even a poorly efficacious vaccine (high loss in efficacy over time) was effective in decreasing the number of symptomatic cases (Fig.   4C). However, even a moderate decrease in protection allows for the emergence of a fourth wave in all age groups (Fig. 4B), with a particularly higher increase in the number of infected hosts in the asymptomatic adult group and, to a lesser extent, among the asymptomatic children. A high decrease in vaccine efficacy was consistently associated with the emergence of the fourth wave, with a larger number of infections in all groups, particularly among children and adult hosts. Among the elderly, there was also an increase in the number of symptomatic cases. Severe cases were abundant in the elderly group during the fourth wave. In the case of a maximum decrease in vaccination efficacy, a large number of cases with a high proportion of symptomatic and severe cases occurred in the adult and elderly groups.

### Effects of elderly-only vaccination, with and without boosters

Figure 5 presents the effects of elderly-only vaccination (EOV) represented in a time length of 25 000 steps (2.85 years). The 4 panels in the upper part of Fig. 5A show the results of non-vaccination. The two 4-panel sections below Fig. 5A show the effects of EOV, without (Fig. 5B) and with (Fig. 5C) booster (third double dose). The number of infected cases in the children and adolescents group did not change due to EOV.

The effect of EOV on mortality (total deaths over the course of 2.85 years) from the start of the epidemic was analyzed in the various age groups, compared with universal vaccination (UNV; all age groups) or no vaccination (NOV). No mortality was detected for the children (0–12 years) in any of the above cases. For the adolescent group (13–19 years), there was 1 death (probably a rare stochastic event) in the EOV intervention. In the adult group (20–59 years), there were 71 deaths in the NOV, 61 in the EOV (14.08% reduction in mortality) and 56 in the UNV (21.13% reduction) groups. There was a major effect in the elderly group (>60 years), with 344 deaths registered in NOV, which was reduced to 166 deaths with EOV (50.30% reduction in mortality). In the UNV intervention landscape, deaths among the elderly reduced by only 3 (n = 163) (51.20% reduction when compared with NOV).

These data are global (from the time of the epidemic's emergence); however, the EOV strategy was implemented only at step 9760 (1.11 years) from the start of the epidemic. In this pre-vaccination period, there were 45 deaths in the adult group and 100 in the elderly group. The EOV strategy reduced deaths in the adult group from 26 to 16 (38.46% reduction), and the UNV strategy reduced the deaths from 26 to 11 (57.70%). As could be expected, the maximum efficacy of EOV affected mortality in the elderly. Without vaccination, 234 deaths occurred in this group, which was reduced to 66 (71.79%) with EOV. UNV spared only 3 of these deaths (73.08% reduction).

Booster vaccines enhance waning immunity against SARS-CoV-2 in previously vaccinated individuals (Atmar et al. 2022). In our simulation, after completing a full vaccination with a *vaccine with moderate loss of efficacy* over time, a booster shot with a vaccine with a moderate loss of efficacy (6 months) significantly reduced the number of infected hosts and shortened the duration of the fourth wave but did not prevent a fifth wave, particularly among asymptomatic children and adults.

Only the booster vaccine implemented in the elderly group caused a certain reduction in the number of infections in the adult E-inn/N-acq (N) (8.65% reduction) and I-inn/N-acq (S) (7.53% reduction) groups (these reductions corresponded to the fifth wave). The effects of EOV almost completely suppressed the fourth and fifth waves in the elderly patient group.

A booster shot with a long-term efficacious vaccine strongly reduces the number of infected patients, completely suppressing the fifth wave in all host groups, except for a few cases in the asymptomatic child population. Even without the booster vaccine, there is a clear long-term reduction in the number of cases in all ages of vaccinated individuals. When focusing only on the effects of booster vaccination on the elderly population (Fig. 5, elongated box at the bottom), there is a clear decrease in the number of infected cases in this group, particularly in the symptomatic and potentially more severe cases (green and blue lines), which is also reflected in an approximately 50% decrease in predicted mortality; however, the number of cases in our population is too low to allow for a representation.

The simulation of the effects of the duration of implementation (4, 6, or 8 months) of the booster shot after the full vaccination schedule is shown in Fig. 6. There was no clear difference when comparing booster shots provided at 4 and 6 months. In all scenarios, boosting the dose suppressed the prolonged tail of low numbers of cases in all age groups except for non-symptomatic adults, however, the number of infected hosts in this group decreased with early booster intervention compared with and late (8 months) intervention.

### Elderly-only vaccination in combination with interventions to decrease transmission

We compared 3 scenarios: (i) lockdown applied only to individuals ≥60 years old for 2 months starting 45 days after the start of the epidemic; the members of this group stopped attending elderly day centers and social clubs, reduced by 80% their activities that required leaving home, and visits to nursing homes were fully suppressed; the vaccination schedule was maintained but with no other intervention on transmission; (ii) lockdown applied only to elderly individuals (as previously explained); however, interventions to reduce transmission were applied to the entire community (all ages), reducing general transmission by 60% and 95% in the hospitals and elderly nursing homes, respectively; and (iii) generalized lockdown (all ages) and implementation of transmission interventions as in the previous scenario. The comparative effects of elderly-only lockdown with or without transmission-reducing interventions revealed that transmission-reducing interventions had a very small effect on the number of infected cases in the child and adolescent populations and a modest effect on the adult population (except for the first wave); however, the number of elderly cases was reduced (peak prevalence reduced by 34.44%). In the hypothetical third scenario of a generalized lockdown (all ages) and transmission-reducing interventions, the number of infected cases in all ages certainly decreased but, counterintuitively, less so in the elderly population. This result can be explained by the implementation of transmission-reducing interventions, which reduced the duration of the first wave in all populations. The second and third waves therefore occurred earlier, and the vaccination had insufficient time to provide protection, decreasing its protection efficacy, which is reflected in the higher elderly mortality (162 cases vs. 136 deaths, a 16% increase) when general transmission barriers were implemented over a general lockdown (Fig. 8).

## Materials and methods

### Baseline demographic structure of the simulated host population

The demographic structure of the simulated host population challenged by SARS-Cov-2 was detailed in a previous study (Campos et al. 2021). However, a summary of this highly granular analysis is available in Table S1 in the Supplementary Material.

### Vaccination schedule per age group

Vaccination started 13.5 months after the start of the epidemic, after 1.5 months without interventions, followed by 2 months of stringent lockdown, including closure of education facilities, most work places and leisure spaces, 80% reduction of outdoor activities, no visits of relatives to elderly nursing homes, and interventions to decrease community transmission by 60% and transmission in hospitals and elderly facilities by 95%. This period was followed by a period of less stringent lockdown, with closure of leisure spaces and implementation of interventions to reduce community transmission and transmission in hospitals and nursing homes by 90%. The priority for vaccination was by decreasing age group, given the relationship between severity and the age of the infected patients. The vaccine protocol included a first dose (protecting 50% from infection and 50% from symptomatic illness), followed 3 weeks later by a second dose (protecting 95% from contagion and 95% from clinical infection). In our simulation, vaccination started in the first month in the elderly population and progressed by 20% per month, so that after 5 months the entire population was administered the vaccine. The 20–59-year age group (10% of the population) started vaccination at month 5; in successive months, 30%, 50%, and 75% of this group were vaccinated, reaching full vaccination at month 9. The 13–19-year age group started vaccination at month 9 (20%), becoming fully vaccinated by month 12. The 0–12-year age group started vaccination at month 11, achieving full vaccination by month 12.

### Influence of different levels of vaccine efficacy over time

In the first scenario, the two-dose vaccination provides full protection; in the second scenario, vaccination decreases in efficacy after 6 months from the date of maximum protection. The rate of protection loss was qualitatively classified into the following 4 categories, according to the rate of infected individuals and the rate of symptomatic individuals progressing to severe infection: (i) no reduction in protection, when the original protection rate did not change (95%); (ii) moderate reduction in protection, with decreases of 50% and 80%, respectively; (iii) strong reduction, with decreases of 25% and 40%, respectively; and (iv) full loss of vaccine efficacy, when the original 95% protection rates decreased to 5% and 5%, respectively. These categories are based on the consideration that a host has the same probability rates of not being infected when exposed to the virus if administered a vaccine with a reduction in protection of 95%, 80%, 50%, 40%, and 25%.

### Vaccination booster dosing

Our simulation included a vaccine booster (using a vaccine with moderate loss of efficacy, see above) dosing at different times (4, 6, and 8 months after the standard vaccination period, and the results are compared with results in the absence of booster dose application). The booster schedule after vaccination is as follows: in the first month, 50% of elderly individuals are administered a booster vaccine; in the second month, the other 50% of this group is administered the booster; in the third month, 50% of adult individuals are administered the booster; in the fourth month, the rest of this group is administered the booster; in the fifth month, the booster vaccine is administered to 50% of the adolescent population; lastly, in the sixth month, the remaining 50% of adolescents and the entire child population are vaccinated.

## Discussion

The detailed study of SARS-Cov-2 epidemiology requires quantitatively projecting the impact of vaccination, antivirals, and therapeutics in a highly detailed manner, considering the host's heterogeneity in terms of age, immune response, and lifestyle, given that these factors affect the virus’ exposure and transmissibility and the individual's susceptibility to infection (Saad-Roy et al. 2020, Collier et al. 2021). Membrane computing applied to the simulation of epidemics allows for a high granularity dissection of the factor complex, ranging from microbiological to sociological, influencing the dynamics and outcomes of these processes. Applied to the case of the SARS-Cov-2 epidemic, our previous study (Campos et al. 2021) focused on non-pharmacological interventions, indicating the possibilities of this predictive computing technology. The main advantage of membrane computing is its ability to simulate (independently and in combination) the possible effects of interventions in halting epidemic processes. The present study considered the effects of vaccination on the dynamics of SARS-Cov-2 epidemics in a particular baseline landscape, simulating a town of approximately 10 000 inhabitants exposed to the virus over several years. The model can be easily modified ‘à la carte’, allowing users to establish numerous and varied conditions and interventions. This study presents just one of these possible landscapes but adjusted to local real-life conditions, as was developed in the previous study (Campos et al. 2021). This study only addresses the effects of vaccination, without considering other possible concomitant interventions, which can be added to the model.

In accordance with other modelling studies (Saad-Roy et al. 2020), our simulation of consecutive waves in the natural epidemic process (Fig. 1) suggests that delayed waves might be larger, because of the accumulation of susceptible hosts through demographics or the waning of immunity. Antibody levels resulting from SARS-Cov-2 natural infection can wane and be insufficient for protection a few months after the challenge; however, protection depends on the specific immune response of differing types of patients (Gudbjartsson et al. 2020, Antia and Halloran 2021; Long et al. 2020, 2021), which is the case for other coronaviruses (Saad-Roy et al. 2020). In fact, the efficacy of the immune response to an infection is central to shaping the endemic phase; the shorter the period of full efficacy, the more rapidly successive waves emerge, leading to a type of plateauing in the number of cases over time (Fig. 1). This stabilization of the epidemic curves occurs for the immunological response groups but is dominated by asymptomatic individuals with highly effective innate immunity, presumably leading to a mild immune response, mostly in childhood. A classic SIRS disease transmission model based on differential equations, complemented by the inclusion of components of immune response, also indicates that children lead the endemicity process (Antia and Halloran 2021), probably because they can develop milder (short protection over time) immune responses (Levine et al. 2021, Weisberg et al. 2021). In this work, it was considered that this effect is somewhat compensated by lower transmission rates associated with low viral load in a proportion of asymptomatic children. However, if they are symptomatic, the length of protective acquired immunity was estimated in 5 months. Most children and adults retain high antibody concentrations with virus neutralizing activity for at least 12 months after primary infection (Mensah et al. 2022), and children had a lower risk of reinfection than did adults overall. It has been proposed that the protective time after infection in children can be shorter (Davies et al. 2020). However, reduced periods of immunological protection increase overall hospitalization and mortality, but fatal outcomes in children and teenagers is so low that this parameter can be disregarded in our model with a limited number of individuals (1312 and 848 respectively).

Mathematical models (Makhoul et al. 2020) and clinical and epidemiological evidence (Pritchard et al. 2021) have shown that double SARS-Cov-2 vaccination reduces the number of new SARS-CoV-2 infections, as shown in Fig. 2. We predict that full vaccination of the population with an efficacious vaccine (small loss of protection in time) might prevent the emergence of new waves. As shown in Fig. 3, the largest benefit is for symptomatic patients, mostly elderly, as has been observed in the real world, where the vaccine was highly effective in decreasing mortality (Pritchard et al. 2021). In fact, our results are highly compatible with observational data (Mazagatos et al. 2021). We simulated the effect of vaccinating only the elderly population (Fig. 5), showing a clear decrease in the number of cases in this group (Fig. 5, lower panel, bottom right) and to a certain extent in the adult group, which was probably the result of those adults interacting with the elderly. In the real world, vaccination not only protects vaccinated individuals but also provides cross-protection for unvaccinated individuals (Milman et al. 2021). More importantly, mortality (including overall mortality in the population) significantly decreases when vaccinating only the elderly population, indicating the need for prioritizing this group for vaccination (Fig. 5). These results are in line with the proposal for a more protective vaccination strategy, not considering children and adolescents as significant targets for vaccination (Giubilini et al. 2021). In conclusion, these exercises endorse the roll-out of focused protection of those individuals in the most vulnerable categories, supporting the basic principle of the Great Barrington Declaration (Kulldorff et al 2020).

Several types of SARS-Cov-2 vaccines are becoming increasingly available (Creech et al. 2021, Kyriakidis et al. 2021) but differ in their level of efficacy over time (Krammer 2021), which can be mostly measured by their comparative duration of protection against infection (Subbarao 2021). Such a parameter should be introduced into modeling exercises that include immunological components (Antia and Halloran 2021). In our simulation, even a poorly efficacious-in-time vaccine might be useful for reducing the number of symptomatic cases, a result consistent with those predicted by other classes of models, showing that imperfect vaccines reduce the clinical severity and transmissibility of subsequent infections (Makhoul et al. 2020, Saad-Roy et al. 2020). Our simulation also suggests that even a moderate decrease in protection does not stop the emergence of a fourth wave in all age groups. Our results for booster vaccination (third dose) (Fig. 7) are highly compatible with those from observational studies (Atmar et al. 2022), particularly with respect to reducing mortality in elderly patients (Barda et al. 2021). Recent studies have shown that a second booster vaccine might indeed be useful for elderly and immunocompromised patients (Arbel et al. 2022, Tanne 2022). Lastly, to illustrate the possibility of using our simulation tool to combine different interventions, the effect of elderly-only vaccination was observed in different landscapes involving transmission-limiting interventions (such as lockdown) in the elderly population only or in a generalized intervention (Fig. 9). Unfortunately, field studies about the effect in transmission of non-pharmaceutical interventions rarely consider the final effects on the most vulnerable individuals in the population (Yang et al. 2021). The comparative effects of elderly-only lockdown with or without transmission-reducing interventions reveal that transmission-reducing interventions have a very small effect on the number of infected cases in child and adolescent populations and a modest effect on the adult population (except for the first wave); however, the number of elderly cases was reduced (peak prevalence reduced by 34.44%). These results indicate the relevance of protecting against transmission and ensuring complete vaccination of elderly individuals as a key intervention in SARS-Cov-2 epidemics, showing that this strategy could be sufficient for sharply decreasing mortality in the affected population.

SARS-Cov-2 models can guide the use of quantitative models in other policy-making areas to help decision making in various scenarios (Eker 2020). Membrane computing models might be particularly relevant for testing assumptions in simulated ‘in-silico’ epidemics and helping to clarify the uncertainties in adopting decisions and implementing interventions.

## Supplementary Material

uqac018_Supplemental_File

## References

[bib1] Antia R , Halloran ME. Transition to endemicity: understanding COVID-19. Immunity. 2021;54:2172–6. 34626549 10.1016/j.immuni.2021.09.019 PMC8461290

[bib2] Arbel A , Sergienko R, Friger M et al. Second booster vaccine and covid-19 mortality in adults 60 to 100 years old. Res Square. 2022; [preprint]

[bib3] Atmar RL , Lyke KE, Deming ME et al. Homologous and heterologous covid-19 booster vaccinations. N Engl J Med. 2022;386:1046–57. 35081293 10.1056/NEJMoa2116414 PMC8820244

[bib4] Baquero F , Campos M, Llorens C et al. P systems in the time of COVID-19. J Membr Comput. 2021;3:246–57.

[bib5] Barda N , Dagan N, Cohen C et al. Effectiveness of a third dose of the BNT162b2 mRNA COVID-19 vaccine for preventing severe outcomes in israel: an observational study. Lancet. 2021;398:2093–100. 34756184 10.1016/S0140-6736(21)02249-2 PMC8555967

[bib6] Campos M , Capilla R, Naya F et al. Simulating multilevel dynamics of antimicrobial resistance in a membrane computing model. MBio. 2019;10:e02460–18. 30696743 10.1128/mBio.02460-18 PMC6355984

[bib7] Campos M , San Millán A, Sempere JM et al. Simulating the influence of conjugative-plasmid kinetic values on the multilevel dynamics of antimicrobial resistance in a membrane computing model. Antimicrob Agents Chemother. 2020;64:1–19. 10.1128/AAC.00593-20 PMC7526830 32457104

[bib8] Campos M , Sempere JM, Galán JC et al. Simulating the impact of non-pharmaceutical interventions limiting transmission in COVID-19 epidemics using a membrane computing model. MicroLife. 2021;2:1–14. 10.1093/femsml/uqab011 PMC8499911 34642663

[bib9] Collier DA , Ferreira IA, Kotagiri P et al. Age-related immune response heterogeneity to SARS-CoV-2 vaccine BNT162b2. Nature. 2021;596:417–22. 34192737 10.1038/s41586-021-03739-1 PMC8373615

[bib10] Cramer EY , Ray EL, Lopez VK et al. Evaluation of individual and ensemble probabilistic forecasts of COVID-19 mortality in the united states. Proc Natl Acad Sci USA. 2022;119:e2113561119. 35394862 10.1073/pnas.2113561119 PMC9169655

[bib11] Creech CB , Walker SC, Samuels RJ. SARS-CoV-2 vaccines. JAMA. 2021;325:1318–20. 33635317 10.1001/jama.2021.3199

[bib12] Davies NG , Klepac P, Liu Y et al. Age-dependent effects in the transmission and control of COVID-19 epidemics. Nature Med. 2020;26:1205–11. 32546824 10.1038/s41591-020-0962-9

[bib13] Eker S . Validity and usefulness of COVID-19 models. Humanit Soc Sci. 2020;7:1–5.

[bib14] Gil-Gil T , Ochoa-Sánchez LE, Baquero F et al. Antibiotic resistance: time of synthesis in a post-genomic age. Comput Struct Biotechnol J. 2021;19:3110–24. 34141134 10.1016/j.csbj.2021.05.034 PMC8181582

[bib15] Giubilini A , Gupta S, Heneghan C. A focused protection vaccination strategy: why we should not target children with COVID-19 vaccination policies. J Med Ethics. 2021;47:565–6. 34233955 10.1136/medethics-2021-107700

[bib16] Gudbjartsson DF , Norddahl GL, Melsted P et al. Humoral immune response to SARS-CoV-2 in iceland. N Engl J Med. 2020;383:1724–34. 32871063 10.1056/NEJMoa2026116 PMC7494247

[bib36_1664336335905] Krammer F . A correlate of protection for SARS-CoV-2 vaccines is urgently needed. Nature Med. 2021;27:1147–1148. 34239135 10.1038/s41591-021-01432-4

[bib17] Kulldorff M , Gupta S, Bhattacharya J. The Great Barrington declaration. 2020. See https://gbdeclaration. org.

[bib18] Kyriakidis NC , López-Cortés A, González EV et al. SARS-CoV-2 vaccines strategies: a comprehensive review of phase 3 candidates. Npj Vaccines. 2021;6:1–17. 33619260 10.1038/s41541-021-00292-w PMC7900244

[bib19] Lavine JS , Bjornstad ON, Antia R. Immunological characteristics govern the transition of COVID-19 to endemicity. Science. 2021;371:741–5. 33436525 10.1126/science.abe6522 PMC7932103

[bib21] Long QX , Jia YJ, Wang X et al. Immune memory in convalescent patients with asymptomatic or mild COVID-19. Cell Discov. 2021;7:18 33767156 10.1038/s41421-021-00250-9 PMC7993859

[bib22] Long QX , Tang XJ, Shi QL et al. Clinical and immunological assessment of asymptomatic SARS-CoV-2 infections. Nat Med. 2020;26:1200–4. 32555424 10.1038/s41591-020-0965-6

[bib23] Makhoul M , Ayoub H, Chemaitelly H et al. Epidemiological impact of SARS-CoV-2 vaccination: mathematical modeling analyses. Vaccines. 2020;8:668. 33182403 10.3390/vaccines8040668 PMC7712303

[bib24] Mazagatos C , Monge S, Olmedo C et al. Effectiveness of mRNA COVID-19 vaccines in preventing SARS-CoV-2 infections and COVID-19 hospitalizations and deaths in elderly long-term care facility residents, spain, weeks 53 2020 to 13 2021. Euro Surveill. 2021;26:pii=2100452 10.2807/1560-7917.ES.2021.26.24.2100452 PMC8212595 34142647

[bib25] Mensah AA , Campbell H, Stowe J et al. Risk of SARS-CoV-2 reinfections in children: a prospective national surveillance study between january, 2020, and july, 2021, in england. Lancet Child Adolesc Health. 2022;6:384–92. 35358491 10.1016/S2352-4642(22)00059-1 PMC8959472

[bib26] Milman O , Yelin I, Aharony N et al. Community-level evidence for SARS-CoV-2 vaccine protection of unvaccinated individuals. Nat Med. 2021;27:1367–9. 34113015 10.1038/s41591-021-01407-5

[bib27] Păun Gh , Rozenberg G, Salomaa A (eds.), The Oxford Handbook of Membrane Computing. Oxford: Oxford University Press, 2010.

[bib28] Pérez-Jimenez MJ , Romero-Jiménez A, Sancho-Caparrini F. Complexity classes in models of cellular computing with membranes. Natur Comput. 2003;2:265–85.

[bib29] Pinotti F , Wikramaratna PS, Obolski U et al. Potential impact of individual exposure histories to endemic human coronaviruses on age-dependent severity of COVID-19. BMC Med. 2021;19:19 33430856 10.1186/s12916-020-01887-1 PMC7801230

[bib30] Pritchard E , Matthews C, Stoesser N et al. Impact of vaccination on new SARS-CoV-2 infections in the united kingdom. Nat Med. 2021;27:1370–8. 34108716 10.1038/s41591-021-01410-w PMC8363500

[bib31] Saad-Roy CM , Wagner CE, Baker RE et al. Immune life history, vaccination, and the dynamics of SARS-CoV-2 over the next 5 years. Science. 2020;370:811–8. 32958581 10.1126/science.abd7343 PMC7857410

[bib32] Subbarao K . The success of SARS-CoV-2 vaccines and challenges ahead. Cell Host Microb. 2021;29:1111–23. 10.1016/j.chom.2021.06.016 PMC8279572 34265245

[bib33] Tanne JH . Covid-19: americans who are over 50 or immunocompromised are advised to have second booster. Brit Med J. 2022;376:o842 35354586 10.1136/bmj.o842

[bib34] Weisberg SP , Connors TJ, Zhu Y et al. Distinct antibody responses to SARS-CoV-2 in children and adults across the COVID-19 clinical spectrum. Nat Immunol. 2021;22:25–31. 33154590 10.1038/s41590-020-00826-9 PMC8136619

[bib35] Yang B , Huang AT, Garcia-Carreras B et al. Effect of specific non-pharmaceutical intervention policies on SARS-CoV-2 transmission in the counties of the united states. Nat Commun. 2021;12:3560. 34117244 10.1038/s41467-021-23865-8 PMC8195990

