# Supplementary material for: Simulating the efficacy of vaccines on the epidemiological dynamics of SARS-CoV-2 in a membrane computing model
Source: Microlife. 2022 Sep 16;3:uqac018. doi: 10.1093/femsml/uqac018 (PMC10117710; doi:10.1093/femsml/uqac018)
Supplement: uqac018_Supplemental_File [file uqac018_supplemental_file.docx]

**TABLE S1.**

**Basic demographic data of the simulated healthy population facing SARS-Cov-2**

The population is composed by 10,260 previously healthy individuals (including 1,312 children, 848 teenagers, 5,590 adult workers, 2,380 retired, 100 in nursing homes, 30 health workers)

**Age groups:**

1. 0 to 12 years old;
2. 13 to 19 years old;
3. Between 20 y 59 years old
4. 60 and over 60 years old
5. As the group 3) but acting as hospital health workers with patient’s attendance (nurses and nurse assistants, doctors), and health workers at Elderly Nursing Homes.

**Living spaces**:

1. **Home**, where a family is living
2. **Working place**, where adults meet in working hours
3. **Children’s School**, where the hosts in the 0-12 years old range meet in school hours
4. **Teenagers School**, where the hosts in the 13-19 years old range meet in school hours
5. **Public Spaces**, as streets, shops, and public transportation
6. **Leisure areas**, including week-end massive street drinking meetings
7. **Elderly Day Center**, where a 40% of elderly host meet daily
8. **Elderly Nursing Home**, where a group of elderly hosts have a permanent stay
9. **Hospital Wards**, attended by patients with severe symptoms,
10. **Intensive Care Unit**, the place in Hospital where critical patients are admitted
11. **Post-ICU Setting**, where patients that have been discharged from ICU stay for a week

**Population structure at homes** (with total numbers)

a) 1 external adult worker, 1 internal adult worker, 2 children less than 12 years old (175)

b) 1 external adult worker, 1 internal adult worker, 1 children less than 12 years old and 1 teen-ager (30)

c) 1 external adult worker, 1 internal adult worker, 2 teen-agers (105)

d) 1 Hospital or Elderly Nursing Home worker, 1 adult home worker, 2 small children (30);

e) 2 home-working elderly people (1190);

f) 1 adult external worker, 1 adult internal worker (496)

g) 1 adult external worker, 1 adult internal worker, 1 small child (276);

h) 1 adult external worker, 1 adult internal worker, 1 teenager (184);

i) 2 external adult workers, 2 children less than 12 years old (175) ;

j) 2 external adult workers 1 children less than 12 years old and 1 teen-ager (30);

k) 2 external adult workers, 2 teen-agers (105);

l) 2 external adult workers, 1 small child (276);

m) 2 external adult workers, 1 teen ager (184);

n) 2 external adult workers (744).

**Division of time in the different hosts.**

1. **for external adult workers**, except Saturday and Sunday, they are from 07:00 to 08:00 in common town space, from 08:00 to 16:00 at the working place, from 17:00 to 19:00 in common space, and from 19:00 to 07:00 at home;
2. **for internal home workers**, starting either at 08:00, 09:00, 10:00, 16:00, 17:00 or 18:00 (10% at each of these times), they have a possibility of been active outside (for instance go shopping) during 1 hour (40%), 2 hours (24%), or 3 hours (36%), every day except Saturday and Sunday;
3. **for children and teen-agers**, they are from 08:00 to 09:00 in common space, 09:00 to 16:00 at the school, and the time to come home again might differ from 17:00 to 18:00 (20%), to 19:00 (48%), to 20:00 (32%); from this time to 08:00 they stay at home, all days except Saturday and Sunday.
4. **for hospital health care workers**, we distinguish day-turn, 07:00 a 08:00 in common space, from 08:00 to 16:00 in hospital wards or ICU, from 17:00 to 19:00 in common space, and from 19:00 a 07:00 at home, and night-turn, from 18:00 to 20:00 in common town space, from 20:00 to 06:00 working in wards or ICU, from 06:00 to 07:00 in common spaces, and from 07:00 to 18:00 at home
5. **Senior Houses** are attended by 20-40% of the retired workers over 60 years old, with a division of time as such: 08:00 a 09:00 common space, 09:00 a 18:00 in the day senior house, and from 18:00-19:00 common space; from 19:00 to 08:00 stay at home.
6. **Elderly Nursing homes**, elderly people live permanently together in a common space, and 5 adult external health workers have a division of time as in hospital health workers.

Other activities are considered. External or internal workers, children and teen-agers might stay during the **week-end** to walk in common spaces, starting a 10:00 (30%), 17:00 (35%) or 18:00 (32.5%) during one hour (40%), two (24%) or three hours (36%). During the dawn and early morning, On Friday night, a high proportion of teen-agers (50 %) and a small proportion of adults (15%) are located in **leisure areas**; on Saturday night the relative proportions are 80% and 30%; they are from 0:00 to 0.01 in common spaces, 01:00 to 06:00 in leisure area, and from 06:00 to 07:00 in common spaces.
